# (*S*)-(+)-*N*-Benzyl­idene-1-(1-naphth­yl)ethyl­amine

**DOI:** 10.1107/S1600536811012980

**Published:** 2011-04-29

**Authors:** Sylvain Bernès, Guadalupe Hernández, Jaime Vázquez, Alejandra Tovar, René Gutiérrez

**Affiliations:** a DEP Facultad de Ciencias Químicas, UANL, Guerrero y Progreso S/N, Col. Treviño, 64570 Monterrey, N.L., Mexico; b Laboratorio de Síntesis de Complejos, Facultad de Ciencias Químicas, Universidad Autónoma de Puebla, A.P. 1067, 72001 Puebla, Pue., Mexico; c Universidad de la Cañada, Cd. Universitaria, 68540, Teotitlán de Flores Magón, Oax., Mexico

## Abstract

In the title chiral aldimine, C_19_H_17_N, the azomethine group is not fully conjugated with the phenyl substituent: the dihedral angle between phenyl and C^*^—N=C mean planes is ϕ_3_ = 23.0 (2)°. Compared with the earlier DFT-B3LYP/6–31 G(*d*) computations from the literature, the C=N—C^*^—C(naph­thyl) torsion angle, found at ϕ_2_ = −118.0 (2)° in the X-ray structure, does not match the angle calculated for the potential minimum energy at ϕ_2_ = 0°. However, this angle is close to the second potential energy minimum at ϕ_2_ = −120° which is *ca*. 8.5 kJ mol^−1^ above the global energy minimum. Thus, the reported X-ray structure corresponds to the second most likely (according to DFT) conformer, allowing the existence of other polymorphs to be anti­cipated.

## Related literature

For a typical synthesis of the title compound, see: Lee & Ahn (2002 ▶). For general background to solvent-free synthesis, see: Tanaka & Toda (2000 ▶). For the structures of related imines, see: Espinosa Leija *et al.* (2009 ▶); Bernès *et al.* (2010 ▶). For the DFT study of the title compound (*R* enanti­omer), see: Fukuda *et al.* (2007 ▶).
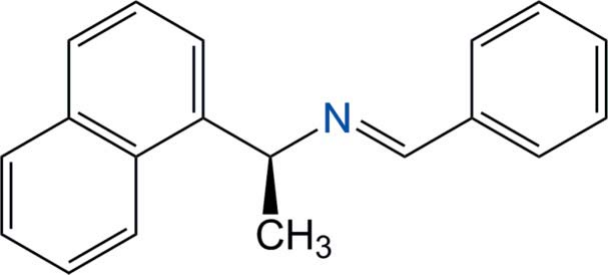


## Experimental

### 

#### Crystal data

C_19_H_17_N
*M* *_r_* = 259.34
Monoclinic, 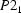

*a* = 8.0761 (8) Å
*b* = 7.7874 (8) Å
*c* = 11.7760 (11) Å
β = 95.033 (7)°
*V* = 737.76 (13) Å^3^
*Z* = 2
Mo *K*α radiation
μ = 0.07 mm^−1^
*T* = 298 K
0.4 × 0.2 × 0.2 mm

#### Data collection

Siemens P4 diffractometer
2470 measured reflections
1595 independent reflections
1276 reflections with *I* > 2σ(*I*)
*R*_int_ = 0.017
3 standard reflections every 97 reflections  intensity decay: 1%

#### Refinement

*R*[*F* ^2^ > 2σ(*F* ^2^)] = 0.034
*wR*(*F* ^2^) = 0.094
*S* = 1.02
1595 reflections
183 parameters
1 restraint
H-atom parameters constrained
Δρ_max_ = 0.09 e Å^−3^
Δρ_min_ = −0.08 e Å^−3^

### 

Data collection: *XSCANS* (Siemens, 1996 ▶); cell refinement: *XSCANS*; data reduction: *XSCANS*; program(s) used to solve structure: *SHELXS97* (Sheldrick, 2008 ▶); program(s) used to refine structure: *SHELXL97* (Sheldrick, 2008 ▶); molecular graphics: *Mercury* (Macrae *et al.*, 2006 ▶); software used to prepare material for publication: *SHELXL97*.

## Supplementary Material

Crystal structure: contains datablocks I, global. DOI: 10.1107/S1600536811012980/ld2008sup1.cif

Structure factors: contains datablocks I. DOI: 10.1107/S1600536811012980/ld2008Isup2.hkl

Additional supplementary materials:  crystallographic information; 3D view; checkCIF report

## Figures and Tables

**Fig. 1.**
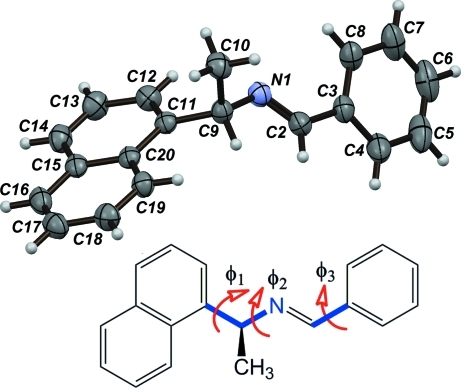
The title molecule with displacement ellipsoids for non-H atoms shown at the 30% probability level. The scheme indicates the torsion angles used in the DFT study of Fukuda et al. (2007).

## supplementary crystallographic information

### Comment

Schiff base compounds are widely studied and used, attracting much attention in both organic synthesis and metal ion complexation. Recently, we have focused our attention on the synthesis of chiral and achiral Schiff bases (Espinosa Leija *et al.*, 2009; Bernès *et al.*, 2010). In continuation of this work, we synthesized the title compound using the solvent-free approach (Tanaka & Toda, 2000). The reaction occurs under mild conditions and requires easier workup procedures and simpler equipment, compared to similar reactions carried out in solution, for example - in refluxing CH_2_Cl_2_ (Lee & Ahn, 2002).

In the title molecule (Fig. 1), all distances and bond angles have expected values. The imine group has a sterically favored *E* conformation, and it is rotated by 23.0 (2)° relative to the phenyl group (the dihedral angle between planes of N1/C2/C9 and C3···C8 groups). The dihedral angle between aromatic phenyl and naphthyl groups is 70.7 (1)°. This molecular conformation is significantly different from one observed in the solid-state for a related imine bearing a thiophene group instead of the phenyl (Espinosa Leija *et al.*, 2009), in which corresponding angles are 5.1 (8) and 83.79 (13)°.

Interestingly, there is a study on conformational flexibility of the title compound that has been published on the basis of DFT calculations at B3LYP/6–31 G(*d*) level (Fukuda *et al.*, 2007). The potential energies for internal rotations around σ bonds C9^*^—C11 (φ_1_), C9^*^—N1 (φ_2_) and C2—C3 (φ_3_) were computed (see Fig. 1 for the angle notations, hereafter assumed for the *S* enantiomer). The dihedral angle φ_1_ related to the orientation of the naphthyl group has two energy minima, with the global minimum at φ_1_ = 40°, close to that found by X-ray diffraction (φ_1_ = N1—C9—C11—C12 = -25.3 (3)°). Similarly, the orientations for the phenyl ring are consistent between DFT and X-ray data: φ_3_ = 0° *vs*. φ_3_ = N1—C2—C3—C8 = 19.9 (4)°. In contrast, internal rotation φ_2_ computed by DFT presents a minimum at φ_2_ = 0°, far different from the angle observed in the crystal structure: φ_2_ = C2—N1—C9—C11 = -118.0 (2)°. However, on the φ_2_ potential curve published by Fukuda *et al.*, there are two lesser minima, at φ_2_ = -120° and φ_2_ = 110°. The first one is consistent with the conformer observed in the solid-state (φ_2_ = -118°) and is placed only 2 kcal/mol above the φ_2_ = 0° minimum. It may thus be expected that the title molecule could be crystallized in different polymorphic phases, derived from conformers with different values for the angle φ_2_.

### Experimental

The title compound was prepared by reacting (*S*)-(–)-(1-naphthyl)ethylamine and benzaldehyde (Lee & Ahn, 2002), but at room temperature and using no solvent. The crude was recrystallized from EtOH affording colorless crystals of the title compound. Yield 94%; mp 79–81 *^o^*C. Analysis: [α]_D_^25^ = +233 (*c* 1, CHCl_3_). FT—IR (KBr): 1641 cm^-1^ (C=N). ^1^H NMR (400 MHz, CDCl_3_/TMS) δ = 1.71 (d, 3H, CH—C*H*_3_,), 5.31 (q, 1H, Ar—C*H*), 7.34–8.24 (m, 12H, Ar), 8.36 (s, 1H, *H*-C=N). ^13^C NMR (100 MHz, CDCl_3_/TMS) δ = 24.51 (C*C*H_3_), 65.51 (*C*HCH_3_), 123.56 (Ar), 123.97 (Ar), 125.26 (Ar), 125.64(Ar), 125.74 (Ar), 127.28 (Ar), 128.21 (Ar), 128.46 (Ar), 128.88 (Ar), 130.52 (Ar), 130.60 (Ar), 133.94 (Ar), 136.43 (Ar),141.12 (Ar), 159.55 (H*C*=N). MS—EI: *m/z*= 259 (*M*^+^).

### Refinement

All C-bonded H atoms were placed in idealized positions and refined as riding to their carrier C atoms, with bond lengths fixed to 0.93 (aromatic CH), 0.96 (methyl CH_3_), and 0.98 Å (methine CH). Isotropic displacement parameters were calculated as *U*_iso_(H) = 1.5*U*_eq_(C10) for the methyl group and *U*_iso_(H) = 1.2*U*_eq_(carrier atom) otherwise. The methyl group C10 was considered as a rigid group but was allowed to rotate about C9—C10 bond. The absolute configuration was assigned from the known configuration of the chiral amine used as the starting material and confirmed by measring the optical rotation and comparing with rotations reported in the litterature for both enantiomers. All measured Friedel pairs (223) were merged.

### Crystal data

**Table:**

C_19_H_17_N	*F*(000) = 276
*M**_r_* = 259.34	*D*_x_ = 1.167 Mg m^−^^3^
Monoclinic, *P*2_1_	Melting point: 352 K
Hall symbol: P 2yb	Mo *K*α radiation, λ = 0.71073 Å
*a* = 8.0761 (8) Å	Cell parameters from 80 reflections
*b* = 7.7874 (8) Å	θ = 4.9–12.3°
*c* = 11.7760 (11) Å	µ = 0.07 mm^−^^1^
β = 95.033 (7)°	*T* = 298 K
*V* = 737.76 (13) Å^3^	Irregular, colourless
*Z* = 2	0.4 × 0.2 × 0.2 mm

### Data collection

**Table:**

Siemens P4 diffractometer	*R*_int_ = 0.017
Radiation source: fine-focus sealed tube	θ_max_ = 26.2°, θ_min_ = 2.5°
graphite	*h* = −10→3
2θ/ω scans	*k* = −1→9
2470 measured reflections	*l* = −14→14
1595 independent reflections	3 standard reflections every 97 reflections
1276 reflections with *I* > 2σ(*I*)	intensity decay: 1%

### Refinement

**Table:**

Refinement on *F*^2^	Secondary atom site location: difference Fourier map
Least-squares matrix: full	Hydrogen site location: inferred from neighbouring sites
*R*[*F*^2^ > 2σ(*F*^2^)] = 0.034	H-atom parameters constrained
*wR*(*F*^2^) = 0.094	*w* = 1/[σ^2^(*F*_o_^2^) + (0.0477*P*)^2^ + 0.0347*P*] where *P* = (*F*_o_^2^ + 2*F*_c_^2^)/3
*S* = 1.02	(Δ/σ)_max_ < 0.001
1595 reflections	Δρ_max_ = 0.09 e Å^−^^3^
183 parameters	Δρ_min_ = −0.08 e Å^−^^3^
1 restraint	Extinction correction: *SHELXL97* (Sheldrick, 2008), Fc^*^=kFc[1+0.001xFc^2^λ^3^/sin(2θ)]^-1/4^
0 constraints	Extinction coefficient: 0.063 (8)
Primary atom site location: structure-invariant direct methods	Absolute structure: 223 Friedel pairs merged

### Fractional atomic coordinates and isotropic or equivalent isotropic displacement parameters (Å^2^)

**Table:**

	*x*	*y*	*z*	*U*_iso_*/*U*_eq_	
N1	0.3971 (2)	0.6059 (3)	0.71942 (14)	0.0646 (5)	
C2	0.4226 (3)	0.6094 (3)	0.61588 (18)	0.0643 (6)	
H2A	0.3363	0.6432	0.5631	0.077*	
C3	0.5833 (3)	0.5624 (3)	0.57416 (18)	0.0662 (6)	
C4	0.6202 (4)	0.6121 (4)	0.4665 (2)	0.0906 (8)	
H4A	0.5440	0.6761	0.4203	0.109*	
C5	0.7722 (5)	0.5659 (5)	0.4274 (3)	0.1090 (11)	
H5A	0.7989	0.6028	0.3561	0.131*	
C6	0.8809 (4)	0.4676 (5)	0.4930 (3)	0.1058 (12)	
H6A	0.9813	0.4356	0.4660	0.127*	
C7	0.8442 (3)	0.4155 (5)	0.5982 (3)	0.0977 (10)	
H7A	0.9188	0.3468	0.6423	0.117*	
C8	0.6973 (3)	0.4640 (4)	0.6394 (2)	0.0748 (7)	
H8A	0.6745	0.4301	0.7121	0.090*	
C9	0.2278 (2)	0.6405 (3)	0.74826 (16)	0.0583 (5)	
H9A	0.1547	0.6558	0.6781	0.070*	
C10	0.1713 (3)	0.4844 (3)	0.8128 (2)	0.0747 (7)	
H10A	0.1743	0.3844	0.7654	0.112*	
H10B	0.0599	0.5025	0.8328	0.112*	
H10C	0.2442	0.4681	0.8809	0.112*	
C11	0.2202 (3)	0.7992 (3)	0.82185 (16)	0.0538 (5)	
C12	0.3561 (3)	0.8538 (3)	0.88883 (18)	0.0648 (6)	
H12A	0.4567	0.7968	0.8856	0.078*	
C13	0.3473 (3)	0.9946 (3)	0.9628 (2)	0.0760 (7)	
H13A	0.4412	1.0271	1.0092	0.091*	
C14	0.2046 (3)	1.0830 (3)	0.96724 (19)	0.0729 (7)	
H14A	0.2011	1.1765	1.0161	0.087*	
C15	0.0608 (3)	1.0349 (3)	0.89846 (17)	0.0595 (5)	
C16	−0.0910 (3)	1.1256 (3)	0.90111 (19)	0.0729 (7)	
H16A	−0.0950	1.2215	0.9478	0.088*	
C17	−0.2301 (3)	1.0758 (4)	0.8373 (2)	0.0776 (7)	
H17A	−0.3285	1.1369	0.8407	0.093*	
C18	−0.2257 (3)	0.9330 (4)	0.7667 (2)	0.0731 (7)	
H18A	−0.3217	0.8987	0.7233	0.088*	
C19	−0.0826 (2)	0.8434 (3)	0.76058 (18)	0.0618 (6)	
H19A	−0.0822	0.7490	0.7122	0.074*	
C20	0.0660 (2)	0.8899 (3)	0.82582 (16)	0.0529 (5)	

### Atomic displacement parameters (Å^2^)

**Table:**

	*U*^11^	*U*^22^	*U*^33^	*U*^12^	*U*^13^	*U*^23^
N1	0.0602 (10)	0.0696 (13)	0.0646 (10)	0.0073 (10)	0.0089 (8)	−0.0043 (10)
C2	0.0694 (13)	0.0571 (13)	0.0670 (12)	0.0072 (12)	0.0095 (11)	−0.0043 (11)
C3	0.0726 (14)	0.0565 (12)	0.0718 (12)	−0.0021 (11)	0.0185 (11)	−0.0127 (11)
C4	0.121 (2)	0.0679 (16)	0.0887 (16)	0.0076 (18)	0.0422 (16)	−0.0017 (14)
C5	0.139 (3)	0.084 (2)	0.116 (2)	−0.017 (2)	0.074 (2)	−0.023 (2)
C6	0.0783 (18)	0.100 (2)	0.145 (3)	−0.0151 (19)	0.0411 (19)	−0.059 (2)
C7	0.0638 (15)	0.112 (3)	0.117 (2)	0.0058 (16)	0.0057 (15)	−0.052 (2)
C8	0.0638 (14)	0.0823 (17)	0.0782 (13)	0.0047 (14)	0.0061 (11)	−0.0211 (13)
C9	0.0544 (11)	0.0602 (12)	0.0606 (11)	0.0038 (11)	0.0073 (9)	−0.0060 (11)
C10	0.0847 (16)	0.0558 (14)	0.0842 (15)	−0.0062 (13)	0.0114 (12)	−0.0021 (13)
C11	0.0571 (11)	0.0528 (12)	0.0530 (10)	−0.0038 (10)	0.0127 (9)	0.0041 (9)
C12	0.0600 (13)	0.0637 (13)	0.0708 (12)	−0.0029 (12)	0.0060 (11)	0.0000 (12)
C13	0.0773 (17)	0.0729 (17)	0.0765 (14)	−0.0175 (15)	−0.0008 (12)	−0.0106 (14)
C14	0.0912 (17)	0.0565 (14)	0.0723 (13)	−0.0121 (13)	0.0155 (12)	−0.0119 (11)
C15	0.0730 (14)	0.0493 (11)	0.0587 (11)	−0.0047 (11)	0.0209 (10)	0.0048 (10)
C16	0.0884 (18)	0.0584 (13)	0.0768 (14)	0.0041 (14)	0.0341 (13)	−0.0024 (13)
C17	0.0715 (16)	0.0747 (17)	0.0901 (15)	0.0131 (14)	0.0269 (13)	0.0072 (14)
C18	0.0605 (13)	0.0809 (17)	0.0794 (14)	0.0022 (13)	0.0145 (11)	0.0021 (13)
C19	0.0601 (13)	0.0612 (13)	0.0655 (11)	−0.0024 (11)	0.0139 (10)	−0.0031 (11)
C20	0.0591 (11)	0.0495 (11)	0.0520 (9)	−0.0052 (9)	0.0166 (8)	0.0045 (9)

### Geometric parameters (Å, °)

**Table:**

N1—C2	1.255 (2)	C10—H10C	0.9600
N1—C9	1.462 (3)	C11—C12	1.362 (3)
C2—C3	1.473 (3)	C11—C20	1.436 (3)
C2—H2A	0.9300	C12—C13	1.405 (3)
C3—C8	1.379 (3)	C12—H12A	0.9300
C3—C4	1.383 (3)	C13—C14	1.348 (3)
C4—C5	1.395 (4)	C13—H13A	0.9300
C4—H4A	0.9300	C14—C15	1.407 (3)
C5—C6	1.355 (5)	C14—H14A	0.9300
C5—H5A	0.9300	C15—C16	1.418 (3)
C6—C7	1.361 (5)	C15—C20	1.420 (3)
C6—H6A	0.9300	C16—C17	1.352 (3)
C7—C8	1.373 (3)	C16—H16A	0.9300
C7—H7A	0.9300	C17—C18	1.391 (4)
C8—H8A	0.9300	C17—H17A	0.9300
C9—C11	1.514 (3)	C18—C19	1.358 (3)
C9—C10	1.525 (3)	C18—H18A	0.9300
C9—H9A	0.9800	C19—C20	1.414 (3)
C10—H10A	0.9600	C19—H19A	0.9300
C10—H10B	0.9600		
			
C2—N1—C9	117.30 (18)	H10A—C10—H10C	109.5
N1—C2—C3	122.9 (2)	H10B—C10—H10C	109.5
N1—C2—H2A	118.5	C12—C11—C20	118.99 (19)
C3—C2—H2A	118.5	C12—C11—C9	121.04 (19)
C8—C3—C4	118.6 (2)	C20—C11—C9	119.90 (18)
C8—C3—C2	121.2 (2)	C11—C12—C13	121.4 (2)
C4—C3—C2	120.2 (2)	C11—C12—H12A	119.3
C3—C4—C5	119.8 (3)	C13—C12—H12A	119.3
C3—C4—H4A	120.1	C14—C13—C12	120.8 (2)
C5—C4—H4A	120.1	C14—C13—H13A	119.6
C6—C5—C4	120.2 (3)	C12—C13—H13A	119.6
C6—C5—H5A	119.9	C13—C14—C15	120.5 (2)
C4—C5—H5A	119.9	C13—C14—H14A	119.8
C5—C6—C7	120.4 (3)	C15—C14—H14A	119.8
C5—C6—H6A	119.8	C14—C15—C16	121.8 (2)
C7—C6—H6A	119.8	C14—C15—C20	119.5 (2)
C6—C7—C8	120.2 (3)	C16—C15—C20	118.7 (2)
C6—C7—H7A	119.9	C17—C16—C15	121.5 (2)
C8—C7—H7A	119.9	C17—C16—H16A	119.2
C7—C8—C3	120.8 (3)	C15—C16—H16A	119.2
C7—C8—H8A	119.6	C16—C17—C18	119.9 (2)
C3—C8—H8A	119.6	C16—C17—H17A	120.1
N1—C9—C11	111.65 (18)	C18—C17—H17A	120.1
N1—C9—C10	107.2 (2)	C19—C18—C17	120.6 (2)
C11—C9—C10	109.68 (15)	C19—C18—H18A	119.7
N1—C9—H9A	109.4	C17—C18—H18A	119.7
C11—C9—H9A	109.4	C18—C19—C20	121.7 (2)
C10—C9—H9A	109.4	C18—C19—H19A	119.2
C9—C10—H10A	109.5	C20—C19—H19A	119.2
C9—C10—H10B	109.5	C19—C20—C15	117.57 (19)
H10A—C10—H10B	109.5	C19—C20—C11	123.62 (18)
C9—C10—H10C	109.5	C15—C20—C11	118.81 (18)
			
C9—N1—C2—C3	−174.6 (2)	C11—C12—C13—C14	−1.9 (4)
N1—C2—C3—C8	19.9 (4)	C12—C13—C14—C15	0.6 (4)
N1—C2—C3—C4	−162.2 (3)	C13—C14—C15—C16	−179.9 (2)
C8—C3—C4—C5	−1.6 (4)	C13—C14—C15—C20	1.4 (3)
C2—C3—C4—C5	−179.5 (2)	C14—C15—C16—C17	−178.0 (2)
C3—C4—C5—C6	2.3 (5)	C20—C15—C16—C17	0.7 (3)
C4—C5—C6—C7	−1.1 (5)	C15—C16—C17—C18	−0.3 (4)
C5—C6—C7—C8	−0.8 (5)	C16—C17—C18—C19	−0.4 (4)
C6—C7—C8—C3	1.6 (4)	C17—C18—C19—C20	0.6 (3)
C4—C3—C8—C7	−0.3 (4)	C18—C19—C20—C15	−0.1 (3)
C2—C3—C8—C7	177.6 (3)	C18—C19—C20—C11	−179.8 (2)
C2—N1—C9—C11	−118.0 (2)	C14—C15—C20—C19	178.3 (2)
C2—N1—C9—C10	121.9 (2)	C16—C15—C20—C19	−0.5 (3)
N1—C9—C11—C12	−25.3 (3)	C14—C15—C20—C11	−2.0 (3)
C10—C9—C11—C12	93.4 (2)	C16—C15—C20—C11	179.16 (18)
N1—C9—C11—C20	157.71 (17)	C12—C11—C20—C19	−179.5 (2)
C10—C9—C11—C20	−83.6 (2)	C9—C11—C20—C19	−2.5 (3)
C20—C11—C12—C13	1.1 (3)	C12—C11—C20—C15	0.8 (3)
C9—C11—C12—C13	−175.92 (19)	C9—C11—C20—C15	177.88 (17)
